# Diallyl Trisulfide Induces Apoptosis in Breast Ductal Carcinoma In Situ Derived and Minimally Invasive Breast Cancer Cells

**DOI:** 10.3390/nu14071455

**Published:** 2022-03-31

**Authors:** Silvia D. Stan, Minna Abtahi

**Affiliations:** Department of Nutrition, College of Agriculture, Biotechnology and Natural Resources, University of Nevada, Reno, NV 89557, USA; abtahiminna@gmail.com

**Keywords:** diallyl trisulfide, *Allium* vegetables, breast ductal carcinoma in situ, apoptosis

## Abstract

Breast ductal carcinoma in situ (DCIS) is a localized form of breast cancer that can progress to invasive breast cancer. Diallyl trisulfide (DATS) is a bioactive compound from *Allium* vegetables reported to induce anticancer effects in several cancer models. The objective of this study was to characterize DATS-induced apoptosis in breast DCIS and minimally invasive breast cancer cells. Breast DCIS cells SUM 102PT (ductal carcinoma in situ with areas of micro-invasion) and SUM 225CWN (chest wall recurrence of ductal carcinoma in situ) were used in this study. DATS induced a dose-dependent reduction in the colony formation ability of breast DCIS cells. DATS inhibited DCIS cell growth by inducing apoptosis as shown by a dose-dependent increase in cytoplasmic histone-associated DNA fragmentation. Induction of apoptosis was more pronounced in SUM 102PT cells than in SUM 225CWN cells at similar concentrations of DATS. DATS-induced apoptosis was characterized by a dose-dependent increase in cleaved poly-ADP ribose polymerase (PARP). DATS treatment resulted in an increase in the cytochrome *c* levels and cleavage of caspases 3, 7, and 9. This study shows that DATS inhibits cell proliferation and induces apoptosis in breast DCIS derived and minimally invasive breast cancer cells, and supports further investigation of DATS as a potential chemopreventive agent for DCIS.

## 1. Introduction

Breast cancer is the most frequently diagnosed type of cancer in women in the United States, with one in eight women being diagnosed with breast cancer during their lifetime [1]. Breast ductal carcinoma in situ (DCIS) is a localized and early stage form of breast cancer that can potentially progress to invasive breast cancer and therefore increase the lifelong risk of developing a lethal breast cancer. DCIS represents approximately 20% of all breast cancer cases in the United States [2,3]. In 2021, it was estimated that 49,290 new cases of breast DCIS would be diagnosed in female patients in the United States [1].

Increased use of mammographic screening for breast cancer has led to an increased number of breast DCIS diagnoses over the last few decades [2,3]. Breast DCIS is often treated aggressively by surgery (mastectomy or breast-conserving surgery) coupled with adjuvant therapies of radiation and endocrine therapy, which brings concerns of potential overtreatment with an unfavorable risk/benefit ratio [3]. Effective and safe chemopreventive strategies for DCIS are still lacking.

DCIS lesions are frequently positive for the expression of human epidermal growth factor receptor 2, HER-2(+), which decreases during the progression from in situ to invasive breast cancer [4]. Similarly, the estrogen receptor (ER) status changes from a predominant ER(+) state in pure DCIS to a lower percentage of ER(+) tumors as DCIS progresses and develops micro-invasions [5,6]. The availability of established breast ductal carcinoma in situ cell lines that resemble the genetic heterogeneity and molecular subtypes of the human disease is limited. In this study, SUM 102PT [ER(−) and HER-2(+)] and SUM 225CWN [ER(−) and HER-2(+)] were used as a model. SUM 102PT cells were isolated from a patient with a tumor consisting mostly of carcinoma in situ with a micro-invasive component [7]. Sum 102PT overexpress HER-2 at both the RNA and protein levels, and expresses constitutively active STAT-3 that is not detected in MCF-10A human epithelial cells cultured under similar conditions [7]. Although negative for cytokeratin-19, SUM 102PT cells are positive for cytokeratins-8 and -18 [7]. SUM 102PT are minimally invasive and have been used as a cell model of the early stages of breast cancer [7,8]. SUM 225CWN cells were isolated from a patient with a chest wall recurrence of ductal carcinoma in situ [8]. CWN stands for chest wall nodule. SUM 225 CWN cells are lowly invasive, and have a luminal subtype [9]. SUM 225CWN cells express cytokeratins-8, -18, and -19.

Epidemiological observations have suggested an inverse association between the consumption of *Allium* vegetables, in particular garlic (*Allium sativum*) and onion (*Allium cepa*), and a lower risk of developing various types of cancer, including breast cancer [10,11,12,13,14,15,16,17]. For example, a case-control study investigating the role of diet on breast cancer risk reported an odds ratio of 0.52 (95% CI: 0.34–0.78) for women consuming 7–10 weekly servings of *Allium* vegetables when compared to women consuming less than 6 weekly servings [12]. *Allium* vegetables contain several bioactive organosulfur compounds, including diallyl trisulfide (DATS), which has been reported to have anticancer effects in several cancer models [10,18,19]. DATS targets multiple cancer hallmark pathways, including induction of cell cycle arrest, induction of apoptosis, inhibition of angiogenesis, generation of reactive oxygen species, activation of c-Jun N-terminal kinase, inhibition of alpha secretases and Notch signaling, and inhibition of angiogenesis, invasion, and metastasis [10,19,20,21]. DATS has been shown to induce ROS-mediated cell cycle arrest and apoptosis and to activate caspases in several cancer models, including breast cancer [10,22].

The objective of this study was to characterize DATS-induced apoptosis in breast DCIS cells.

## 2. Materials and Methods

### 2.1. Reagents

Diallyl trisulfide (DATS, ≥98% purity) was purchased from Sigma Aldrich (St. Louis, MO, USA). Dimethyl sulfoxide (DMSO) and phosphate-buffered saline (PBS) were obtained from Thermo Fisher Scientific (Waltham, MA, USA). Trypan blue (0.4%, *w*/*v*, in PBS) was purchased from Corning (Manassas, VA, USA), and crystal violet and *N*-acetyl-*L*-cysteine were purchased from Sigma Aldrich. Antibodies against PARP (#9542), cleaved PARP (#5625), Bcl2 (#4223), Bak (#3814), cytochrome *c* (#4272), cleaved caspase 3 (#9664), cleaved caspase 7 (#9491), and cleaved caspase 9 (#52873) were purchased from Cell Signaling Technology (Danvers, MA, USA). Actin antibody (#A5441) was purchased from Sigma Aldrich. Secondary anti-mouse and anti-rabbit antibodies were purchased from Cell Signaling Technology (Danvers, MA, USA).

### 2.2. Cell Culture and Media

Human breast cancer cell lines SUM 102PT [ER(−) and HER-2(+)] and SUM 225CWN [ER(−) and HER-2(+)] were purchased from BioIVT (Detroit, MI, USA). SUM 102PT cells were isolated from a patient with minimally invasive intraductal breast carcinoma [8]. SUM 225CWN cells were isolated from a patient with a chest wall recurrence of ductal carcinoma in situ [8]. SUM 102PT cells were cultured in Ham’s F-12 Nutrient Mix (Thermo Fisher Scientific) supplemented with 1 g/L bovine serum albumin (Sigma Aldrich), 10 ng/mL human epidermal growth factor (Sigma Aldrich), 5 mM ethanolamine (Sigma Aldrich), 10 mM HEPES (Corning), 1 µg/mL hydrocortisone (Stem Cell Technologies, Vancouver, BC, Canada), 5 µg/mL insulin (Sigma Aldrich), 50 nM sodium selenite (Sigma Aldrich), 5 µg/mL human APO-transferrin (Sigma Aldrich), 10 nM triiodo-L-thyronine (Sigma Aldrich), 2% heat-inactivated fetal bovine serum (Genesee Scientific, El Cajon, CA, USA), and 1% penicillin-streptomycin solution (Corning). SUM 225CWN cells were cultured in Ham’s F-12 Nutrient Mix supplemented with 10 mM HEPES, 1 µg/mL hydrocortisone, 5 µg/mL insulin, 5% heat-inactivated fetal bovine serum, and 1% penicillin-streptomycin solution. SUM 102PT and SUM 225CWM cells were cultured at a temperature of 37 °C and an atmosphere of 5% CO_2_.

### 2.3. Cell Survival Assay

Cell survival assay was performed by trypan blue dye exclusion assay as described previously [19,23]. Briefly, SUM 102PT and SUM 225CWN cells were plated in 12-well plates (5 × 10^4^ cells/well), allowed to attach for 24 h, and then treated with vehicle (control) or DATS (50, 100, and 200 µM) for 24 h. Viable cells were quantified after trypan blue staining using a hemocytometer.

### 2.4. Clonogenic Assay

Colony formation was determined using a clonogenic assay as described previously [19]. Briefly, SUM 102PT and SUM 225CWN cells were cultured in 100 mm plates with 1 × 10^6^ cells/plate and incubated for 24 h for cell attachment. Cells were then treated with the indicated concentrations of DATS or vehicle (control, DMSO/PBS) for 24 h. After treatment, SUM 102PT and SUM 225CWN cells were reseeded at equal densities into 6-well plates with 300 (SUM 102PT) or 1000 cells/well (SUM 225CWN), and incubated in the absence of DATS for 11 (SUM 102PT) or 28 days (SUM 225CWN). Cell growth medium was replaced every 2–3 days during the incubation periods. Colonies were washed with PBS, fixed with ice-cold methanol, and stained with 0.5% (*w*/*v*) crystal violet before counting.

### 2.5. Apoptosis Assay

Apoptosis was quantified using a cell death detection ELISA^PLUS^ kit from Roche (Pleasanton, CA, USA). SUM 102PT and SUM 225CWN cells were plated in 12-well plates with 5 × 10^4^ cells/well and incubated for 24 h for cell attachment. Cells were then treated with the indicated concentrations of DATS or vehicle (control) for 24 h. Apoptosis was determined by quantifying the cytoplasmic histone-complexed DNA fragments using the photometric enzyme immunoassay Cell Death Detection ELISA^PLUS^ kit, according to the manufacturer’s instructions.

### 2.6. Western Blotting

SUM 102PT and SUM 225CWN cells were cultured in 100 mm plates with 1 × 10^6^ cells/plate and incubated for 24 h for cell attachment. Cells were then treated with the indicated concentrations of DATS or vehicle (control) for 24 h. Cells pellets were collected and subsequently lysed with lysis buffer (Cell Signaling Technology) supplemented with protease and phosphatase inhibitors (Sigma Aldrich). The protein concentration was determined using a Bradford protein assay (Bio-Rad, Hercules, CA, USA). The proteins were resolved on 10% or 12% SDS-polyacrylamide gels prepared using the TGX FastCast kit (Bio-Rad) and then transferred to a PVDF membrane using the Trans-Blot Turbo Mini-PVDF pack (Bio-Rad). The membrane was blocked for 2 h at room temperature in 5% (*w*/*v*) nonfat dry milk in TTBS, and then incubated with the appropriate primary antibody overnight at 4 °C. Subsequently, the membrane was washed with TTBS, incubated with the appropriate secondary antibody for 1 h at room temperature, washed again with TTBS, and then developed using SuperSignal^TM^ West Femto maximum sensitivity substrate (Thermo Scientific, Rockford, IL, USA). Protein bands were detected and visualized using the ChemiDoc Imaging System with Image Lab Software (Bio-Rad).

### 2.7. Statistical Analysis

Statistical analysis was completed using GraphPad Prism 8.0 software (San Diego, CA, USA). One-way ANOVA followed by Dunnett’s or Tuckey’s multiple comparison tests, as appropriate, were used to determine differences between groups. *p* values < 0.05 were considered significant.

## 3. Results

### 3.1. Diallyl Trisulfide Decreases the Survival of Breast Ductal Carcinoma In Situ Cells

**Figure 1:**
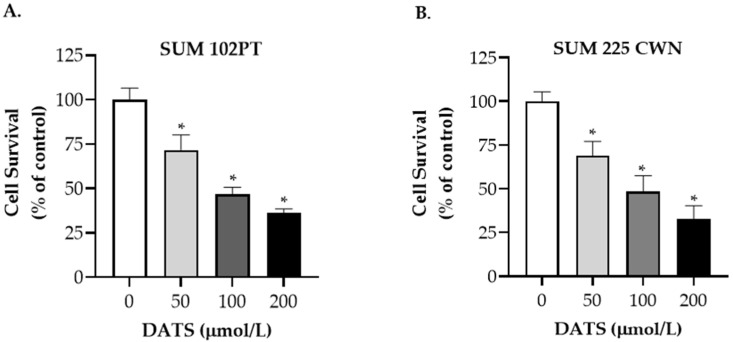
Effect of diallyl trisulfide (DATS) on the survival of DCIS cells. (**A**) Effect of DATS treatment on the survival of SUM 102PT cells as determined by trypan blue dye exclusion assay after a 24-h treatment. Columns, mean; bars, SD of three independent experiments. *, *p* < 0.05, significantly different by 1-way ANOVA followed by Dunnetts’s multiple comparisons test. (**B**) Effect of DATS treatment on the survival of SUM 225CWN cells as determined by trypan blue dye exclusion assay after a 24-h treatment. Columns, mean; bars, SD of three independent experiments. *, *p* < 0.05, significantly different by 1-way ANOVA followed by Dunnetts’s multiple comparisons test.

We first examined the effect of DATS on the survival of DCIS cells. Cells were treated with DMSO (control) or DATS (50, 100, 200 µmol/L) for 24 h and cell survival was quantified by trypan blue dye exclusion assay. Treatment with DATS induced a dose-dependent inhibition of the survival of SUM 102PT cells (Figure 1A) and SUM 225 CWN cells (Figure 1B), indicating IC_50_ values of DATS between 90 and 100 µmol/L.

### 3.2. Diallyl Trisulfide Decreases the Colony Formation Ability of Breast Ductal Carcinoma In Situ Cells

**Figure 2:**
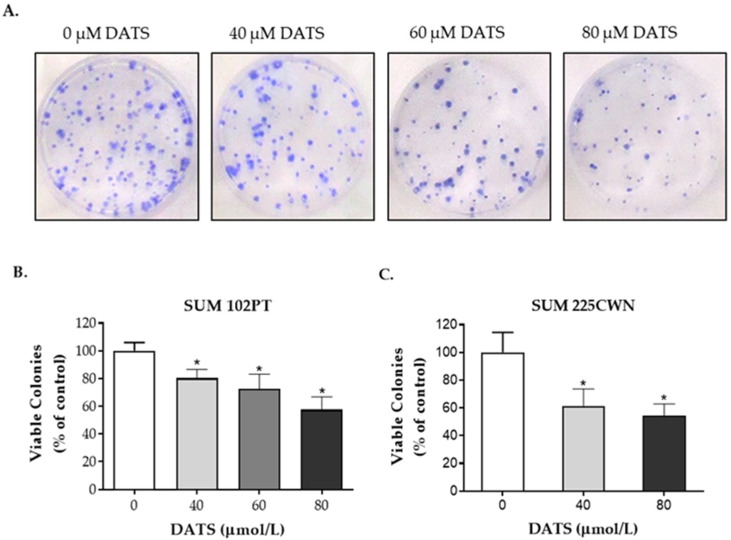
Effect of DATS on the colony formation of DCIS cells. (**A**) Representative wells showing colony formation in SUM 102PT cells. (**B**) Quantification of the colony formation of SUM 102PT cells. (**C**) Quantification of the colony formation of SUM 225CWN cells. Columns, mean; bars, SD of three independent experiments. *, *p* < 0.05, significantly different in comparison with vehicle-treated (control) cells by 1-way ANOVA followed by Dunnetts’s multiple comparisons test.

The effect of DATS on the colony formation of DCIS cells is shown in Figure 2. SUM 102PT cells were treated with DMSO (control) or DATS (40, 60, 80 μmol/L) for 24 h, reseeded at equal densities in the absence of DATS, and then incubated for 11 days before staining and counting. SUM 225CWN cells were treated with DMSO (control) or DATS (40, 80 μmol/L) for 24 h, reseeded at equal densities in the absence of DATS, and then incubated for 28 days. DATS-treated cells showed a dose-dependent reduction in their colony formation ability, suggesting a lasting effect of DATS on the cell proliferation of DCIS cells after DATS removal. Colony formation was significantly inhibited by 42% and 46% after the 80 μmol/L DATS treatment in SUM 102 PT and SUM 225CWN cells, respectively (Figure 2B,C).

### 3.3. Diallyl Trisulfide Induces Apoptosis in Breast Ductal Carcinoma In Situ Cells

**Figure 3:**
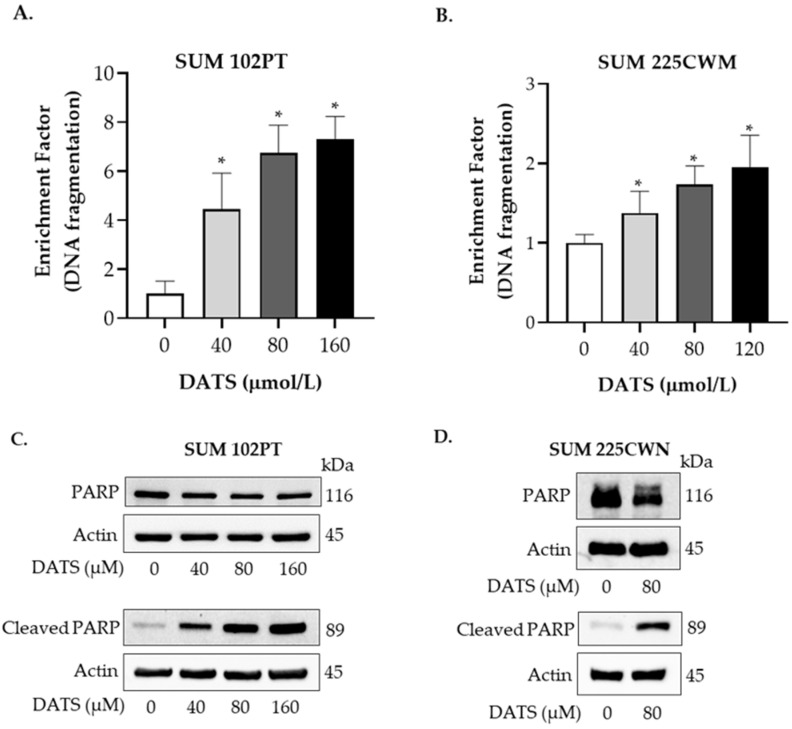
DATS induces apoptosis in DCIS cells. (**A**) Quantification of cytoplasmic histone-associated DNA fragmentation in SUM 102PT cells after exposure to vehicle (control) or various concentrations of DATS for 24 h. DNA fragmentation enrichment factor relative to the corresponding control is shown. Columns, mean; bars, SD of three independent experiments. (**B**) Quantification of cytoplasmic histone-associated DNA fragmentation in SUM 225CWN cells after exposure to vehicle (control) or various concentrations of DATS for 24 h. Columns, mean; bars, SD of three independent experiments. *, *p* < 0.05, significantly different in comparison with control by 1-way ANOVA followed by Dunnett’s multiple comparisons test. Western immunoblotting for PARP and cleaved PARP using lysates from SUM 102PT cells (**C**) and SUM 225CWN cells (**D**) after exposure to vehicle (control) or DATS for 24 h.

Apoptosis was quantified using a cell death detection ELISA^PLUS^ kit from Roche according to the manufacturer’s instructions. SUM 102PT cells were treated with DATS (40, 80, 160 μmol/L) or vehicle (control) for 24 h and then quantified for cytoplasmic DNA fragments. Quantification of the cytoplasmic histone-associated DNA fragmentation in DCIS cells is shown in Figure 3A (SUM 102PT) and Figure 3B (SUM 225CWN). Treatment with DATS resulted in a dose-dependent increase in the induction of apoptosis in DCIS breast cancer cells. Induction of DNA fragmentation was more pronounced in SUM 102PT cells than in SUM 225CWN cells at similar concentrations of DATS.

### 3.4. Diallyl Trisulfide Modulates the Expression of Apoptotic Markers in Breast Ductal Carcinoma In Situ Cells

Western immunoblotting was used to determine the effect of DATS on apoptosis molecular markers. Cleavage of poly-ADP ribose polymerase (PARP) represents a hallmark of cells undergoing apoptosis. PARP is synthesized as a 116 kDa nuclear protein, which is cleaved during apoptosis by executioner caspase 3 to yield an 89 kDa protein. Treatment with DATS resulted in an increase in cleaved PARP in SUM 102 PT cells (Figure 3C) and SUM 225 CWN cells (Figure 3D). A dose-dependent increase in cleaved PARP was observed after a 24-h treatment in SUM 102PT cells (Figure 3C). This effect was associated with a modest decrease in total PARP. Probing with β-actin antibody was used as a protein loading control.

Apoptotic stimuli provoke the permealization of mitochondrial membranes [24]. In response to apoptotic stimuli, B cell lymphoma 2 (Bcl2) protein family members translocate to the mitochondria and regulate apoptosis by interfering with the release of apoptogenic proteins into the cytosol [25]. Mitochondrial membrane integrity is regulated by opposing actions of the pro-apoptotic members of the Bcl2 family, such as Bak and Bax, and anti-apoptotic members of the Bcl2 family, such as Bcl2 and Bcl-xL.

**Figure 4:**
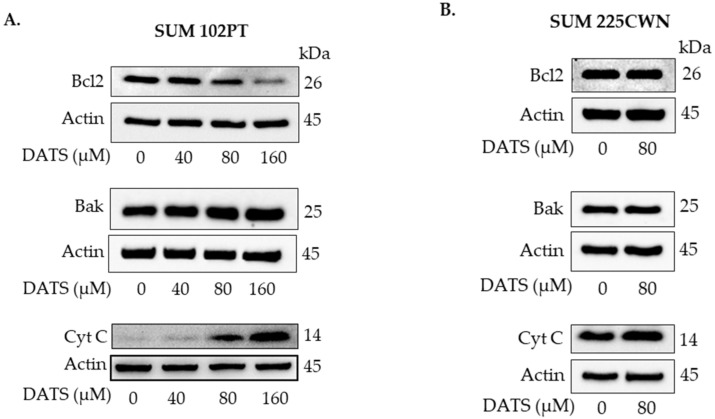
Western immunoblotting for Bcl2, Bak, and cytochrome *c* using lysates from SUM 102PT cells (**A**) and SUM 225CWN cells (**B**) after exposure to vehicle (control) or the indicated concentrations of DATS for 24 h.

In SUM 102PT cells exposed to DATS, the expression levels of anti-apoptotic protein Bcl2 were decreased and cytochrome *c* levels were increased in a dose-dependent manner (Figure 4A). The effect was less pronounced in SUM 225CWN cells (Figure 4B). The expression levels of pro-apoptotic protein Bak (Bcl2 homologous antagonist) were only minimally affected by DATS treatment (Figure 4A).

### 3.5. Diallyl Trisulfide Induces Cleavage of Caspases 3, 7, and 9 in Breast Ductal Carcinoma In Situ Cells

Caspases are aspartate-specific cysteine proteases that play a vital role in apoptosis. Cleavage of procaspase forms leads to the activation of caspases and can occur through an intrinsic (mitochondrial) pathway and an extrinsic (death receptor) pathway.

**Figure 5:**
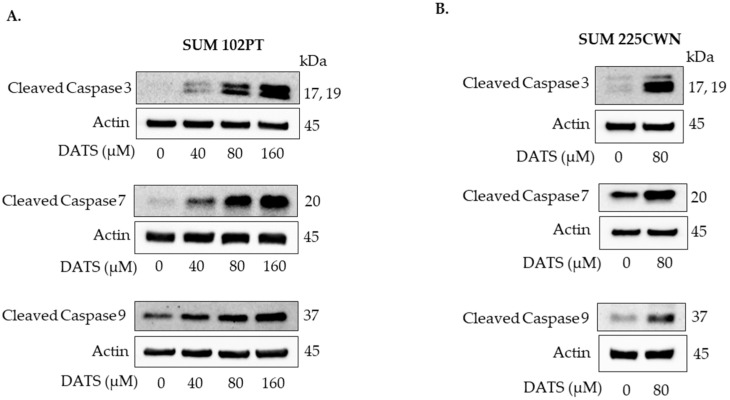
Western immunoblotting for cleaved caspases 3, 7, and 9 using lysates from SUM 102PT cells (**A**) and SUM 225CWN cells (**B**) after exposure to vehicle (control) or the indicated concentrations of DATS for 24 h.

DATS treatment resulted in cleavage of caspases 3, 7, and 9 in both SUM 102PT (Figure 5A) and SUM 225CWN cells (Figure 5B).

### 3.6. Diallyl Trisulfide Induces ROS-Dependent Apoptosis in Breast Ductal Carcinoma In Situ Cells

**Figure 6:**
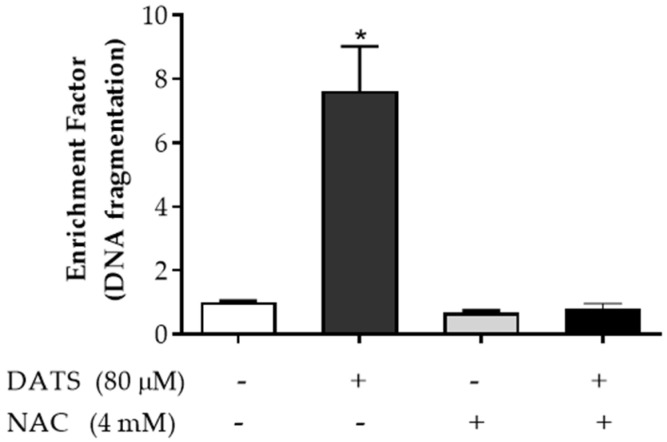
Effect of ROS scavenger *N*-acetyl-*L*-cysteine (NAC) treatment on DNA fragmentation in SUM 102PT cells. +, −, Cells were pretreated with NAC (4 mM) for 2 h before treatment with NAC (4 mM) and/or DATS (80 µM) for 24 h. Columns, mean; bars, SD of three independent experiments. *, *p* < 0.05, significantly different by 1-way ANOVA followed by Tuckey’s multiple comparisons test.

We next sought to determine if DATS-induced apoptosis is mediated by the generation of reactive oxygen species (ROS). The effect of the general antioxidant *N*-acetyl-*L*-cysteine (NAC) treatment on cytoplasmic histone-associated DNA fragmentation in SUM 102PT cells is shown in Figure 6. DATS-induced apoptosis was abrogated by treatment with the ROS scavenger NAC, suggesting an ROS-mediated apoptosis in DCIS cells.

## 4. Discussion

Breast DCIS is a localized and early stage form of breast cancer that can potentially progress to an invasive breast cancer phenotype, although it is not currently possible to predict which patients with ductal carcinoma in situ will progress clinically to a metastatic form of breast cancer and which patients will not [2,26]. The potential progression to an invasive form of breast cancer increases the lifelong risk of developing a lethal breast cancer and creates a need for novel prevention strategies against this disease.

Diallyl trisulfide is a bioactive organosulfur compound, derived from *Allium* vegetables, that has been reported to have multiple anticancer effects. DATS has been shown to induce ROS-mediated cell cycle arrest and apoptosis and to activate caspases in several cancer models ([10], and references therein). However, the effect of DATS on DCIS has not been reported. In the present study, we showed that DATS inhibits the cell proliferation and colony formation ability of breast DCIS cells. The presence of fragmented DNA indicates induction of apoptosis. Western blot analysis implicates mitochondria in DATS-induced apoptosis of DCIS cells. DATS inhibits DCIS growth by inducing caspase-mediated intrinsic (mitochondrial pathway) apoptosis in breast DCIS cells.

Mitochondria regulate apoptotic cell death. Apoptotic stimuli, such as DNA damage, provoke the permeabilization of mitochondrial membranes and trigger mitochondria to cause the release of cytochrome *c* into the cytosol [24,27]. In the cytosol, cytochrome *c* binds the cytosolic molecule Apaf-1 (apoptotic protease activating factor-1), and induces a conformational change in Apaf-1 that allows the binding of dATP or ATP, and subsequently leads to the formation of an apoptosome, an Apaf-1 and cytochrome *c* complex [24,25]. The apoptosome recruits the initiator pro-caspase 9 and triggers its activation. Activated caspase 9 cleaves and activates executioner caspases, such as caspases 3 and 7 [25]. Subsequently, executioner caspases cleave additional intracellular substrates, leading to the morphological changes characteristic of apoptosis, such as nucleosomal DNA fragmentation, chromatin condensation, and nuclear membrane breakdown [24,25].

Our study showed that DATS treatment results in an increase in cytochrome *c* and cleavage of caspases 9, 3, and 7 in DCIS derived and minimally invasive breast cancer cells. Caspases (cysteine aspartate-specific proteases) are a class of cysteine proteases activated during apoptosis [24]. Both the intrinsic pathway of apoptosis (also called mitochondrial) and the extrinsic pathway of apoptosis (also called death receptor) lead to activation of executioner caspase 3 and caspase 7 [27]. The results of this study show that DATS-induced apoptosis in SUM 102PT and SUM225CWN cells is mediated by the initiator caspase 9 and executioner caspases 3 and 7.

Activated executioner caspases will subsequently cleave several downstream substrates, leading to the characteristic morphological changes of apoptosis induction. A hallmark of cells undergoing apoptosis is cleavage of PARP. This study showed that DATS induces proteolytic cleavage of PARP in DCIS cells.

The generation of ROS is known to induce cell death. DATS has been shown to induce cell cycle arrest and apoptosis by enhancing the generation of ROS [10]. The present study showed that DATS-induced apoptosis in DCIS cells was abolished by ROS scavenger (NAC) treatment, suggesting the induction of ROS-dependent apoptosis in DCIS cells.

## 5. Conclusions

Alternative strategies, including cancer chemopreventive strategies, are needed to address the possibility of overtreatment in the management of breast DCIS. Inducing apoptosis in early stage forms of breast cancer may be an effective strategy to prevent DCIS progression to more invasive phenotypes. This study showed that DATS, a bioactive compound derived from *Allium* vegetables, inhibits the cell proliferation and colony formation ability of DCIS derived and minimally invasive breast cancer cells, and induces dose-dependent apoptosis. DATS-induced apoptosis is mediated by ROS generation and occurs through activation of the initiator caspase 9 and executioner caspases 3 and 7. This study supports further investigation of *Allium*-derived compounds as potential chemopreventive agents for DCIS. The development of additional in vitro and in vivo models of DCIS would help characterize the anticancer and potential chemopreventive effects of natural agents, such as DATS.

## Data Availability

Not applicable.
